# Insight in miRNome of Long-Term Non-Progressors and Elite Controllers Exposes Potential RNAi Role in Restraining HIV-1 Infection

**DOI:** 10.3390/jcm9082452

**Published:** 2020-07-31

**Authors:** Rubén Ayala-Suárez, Francisco Díez-Fuertes, Esther Calonge, Humberto Erick De La Torre Tarazona, María Gracia-Ruíz de Alda, Laura Capa, José Alcamí

**Affiliations:** 1 AIDS Immunopathology Unit, National Center for Microbiology, Institute of Health Carlos III, Majadahonda, 28220 Madrid, Spain; rayala@isciii.es (R.A.-S.); ecalonge@isciii.es (E.C.); erickdlt@isciii.es (H.E.D.L.T.T.); lcapa@isciii.es (L.C.); 2 HIV Unit, Hospital Clínic de Barcelona, 08036 Barcelona, Spain; 3 Sección de Enfermedades Infecciosas, Medicina Interna, Complejo Hospitalario de Navarra, 31008 Pamplona, Spain; mgraciaruizdealda@gmail.com

**Keywords:** HIV-1 infection, long-term non-progressors, elite controllers, miRNA-Seq, miRNome, differential expression, biomarker discovery

## Abstract

Long-term non-progressors (LTNP) and elite controllers (EC) represent spontaneous natural models of efficient HIV-1 response in the absence of treatment. The main purposes of this work are to describe the miRNome of HIV-1 infected patients with different extreme phenotypes and identify potentially altered pathways regulated by differentially expressed (DE) miRNAs. The miRNomes from peripheral blood mononuclear cells (PBMCs) of dual phenotype EC-LTNP or LTNP with detectable viremia and HIV-infected patients with typical progression before and after treatment, were obtained through miRNA-Seq and compared among them. The administration of treatment produces 18 DE miRNAs in typical progressors. LTNP condition shows 14 DE miRNA when compared to typical progressors, allowing LTNP phenotype differentiation. A set of four miRNAs: miR-144-3p, miR-18a-5p, miR-451a, and miR-324 is strongly downregulated in LTNP and related to protein regulation as AKT, mTOR, ERK or IKK, involved in immune response pathways. Deregulation of 28 miRNA is observed between EC-LTNP and viremic-LTNP, including previously described anti-HIV miRNAs: miR-29a, associated with LTNP phenotype, and miR-155, targeting different pre-integration complexes such as ADAM10 and TNPO3. A holistic perspective of the changes observed in the miRNome of patients with different phenotypes of HIV-control and non-progression is provided.

## 1. Introduction

HIV natural infection is characterized by continued viral replication, systemic chronic immune activation, and a strong decline of CD4+ T cells. In most patients, AIDS is developed within a few years from primoinfection, although a small fraction of patients progress to the disease in a smaller amount of time. The introduction of antiretroviral therapy (ART) allowed the control of AIDS manifestation, but HIV infection remains incurable [1]. On that basis, unraveling new mechanisms to control the infection is of the essence to develop new therapeutic approaches in chasing a functional cure. In the past decade, long-term non-progressors (LTNP) and elite controllers (EC) have been extensively studied, due to their ability to maintain high CD4+ T cell levels and undetectable viral load in plasma, respectively, for a long time in the absence of ART [2]. Due to the heterogeneity of these populations, it has been difficult to achieve a consensus definition for these extreme phenotypes. Even so, it is widely considered that both phenotypes represent excellent natural model scenarios of immune control of viral infection.

Long-term non-progression and viremia control are multifactorial circumstances based on viral fitness and immuno-genetic factors. A low proportion of these patients are associated with attenuated or defective viruses that present mutations in viral genes [3,4,5]. Replication-competent HIV-1 has been isolated from EC, suggesting that host factors are crucial in the control of the infection [6]. Among host factors involved in slow progression, the presence of “protective” HLA alleles (HLA-B*57 and HLA-B*27), as well as several polymorphisms inside and outside the HLA region have been described [7,8,9]. The immune response observed in ECs/LTNPs has specific features: higher expression of INF-γ by NK [10], polyclonal and intensified Gag-specific response in CD4+ T cells [11], and a greater production of IL-2, IFN- γ, MIP-1β, TNF-α and granzyme B by CD8+ T cells [12]. Pereyra F, et al. [7] outline that the relevant markers of HLA altogether with protective variants in CCR2 and CCR5 only account for 23% of the observed variance of elite control. Hence, the scientific community strives to find protective factors that are present in a relevant percentage of ECs/LTNPs.

Some studies have investigated the gene expression profile of these individuals [13,14,15]. They have found differences between ECs/LTNPs and typical progressors (TP), which suggest that gene expression regulation may be associated with the progression of the disease. Different mechanisms including epigenetic and post-transcriptional pathways are involved in the regulation of gene expression. RNA interference (RNAi) is a conserved mechanism in eukaryotic cells composed of various small non-coding RNAs, which repress mRNA expression [16]. One of the most important families of RNAi is micro-RNAs (miRNAs), and the interest in their role in viral infection has increased in the last few years [17]. The mature form of miRNAs consists of an asymmetric dsRNA of 19–22 nt which associates in the cytoplasm with various proteins to build RNA-induced silencing complex (RISC), remaining one strand (guide strand) attached to RISC and leading the complex to its target sequences. The degree of complementarity of the miRNA with the 3′ untranslated region (3′-UTR) of the target gene’s mRNA determines if the targeted mRNA is degraded or retained from ribosomal interaction [18]. A single miRNA may target multiple gene transcripts and a single mRNA can be targeted by various miRNAs. Modulation of HIV replication by miRNAs can be exerted in two ways: either directly targeting HIV mRNA or targeting the mRNAs that encode relevant host cell factors that are involved in HIV replication including genes that contribute to immune responses [19]. Some findings show that HIV-1 displays several means to hinder host mechanisms based on RNA interference, which suggests a potential activity of miRNA in viral suppression. 

The study of miRNA expression profiles has provided novel strategies to diagnose, to make prognosis, and even to select a concrete treatment for multiple infectious diseases [20,21,22,23]. The present study aims to identify a miRNA expression pattern associated with ECs/LTNPs phenotypes and through the study of their target genes, to get a better insight into the molecular mechanisms implied in limiting viral replication and disease progression. The analysis of the miRNome of ECs/LTNPs has allowed the identification of deregulated miRNAs and the characterization of their potential target routes involved in the immune control of the HIV-1 replicative cycle.

## 2. Experimental Section

### 2.1. Samples

Peripheral blood mononuclear cells (PBMC) pellets were kindly provided by HIV BioBank integrated into the Spanish HIV/AIDS Research Network (RIS) [24]. Peripheral blood mononuclear cells (PBMC) were isolated on a Ficoll-Hypaque density gradient (Comercial Rafer S.L., Zaragoza, Spain) by the staff of the Spanish HIV BioBank following a Standard Operating Procedure (SOP) [25]. Samples were processed following current procedures and frozen immediately after their reception. In all cases patients’ informed consent was obtained and protocols were approved by the Institutional Ethical Committee (Instituto de Salud Carlos III. CEI PI 10_2011v3). A total of 30 samples were included in the study, 16 HIV-infected subjects classified as long term non-progressors (LTNP) from the Spanish LTNP-RIS cohort and 14 paired samples coming from 7 HIV-positive patients with a typical progression (TP) pattern from the Spanish AIDS Research Network cohort (CoRIS), 7 before receiving antiretroviral therapy (pre-TP) and their counterparts after the administration of ART (post-TP). All experiments were performed in accordance with relevant guidelines and regulations.

### 2.2. Small RNA Extraction

Pellets consisting of 10^7^ PBMCs were received and conserved at −80 °C until their usage. RNA extraction was carried out with AllPrep DNA/RNA/miRNA isolation kit (Qiagen, Hilden, Germany) following the manufacturer’s guidelines. Total RNA quality was assayed in a 2100 Bioanalyzer (Agilent Technologies, Santa Clara, CA, USA) for quality control before DNA library preparation, obtaining a mean RNA integrity number (RIN) of 9.2, this being the value above 8 in all samples. 

### 2.3. Next-Generation Sequencing

The cDNA library was constructed with TruSeq Small RNA library preparation (Illumina, San Diego, CA, USA) following the producer’s recommendations. Quality control of cDNA library was assayed with High Sensitivity DNA kit in Bioanalyzer 2100 (Agilent Technologies, Santa Clara, CA, USA), checking for mature miRNAs plus adaptors expected to be around 150 bp. Samples were pooled together and sequencing was performed on an Hiseq 2000 platform (Illumina, San Diego, USA) with single-end 50 nt reads. All samples were loaded onto one lane of a flow cell. 

### 2.4. Analysis of miRNA

Raw data obtained from sequencing underwent quality analysis through FASTQC software and java tool Trimmomatic v 0.36 [26] was used to trim in every read the adaptors and specific primers generated in cDNA library preparation, allowing a 2 bp maximum discordance. Reads were quality filtered with a 4 bp sliding window and trimmed if the mean of the Phred score of the tetranucleotide fell below 15. Furthermore, reads under 20 bp length were removed in order to avoid potential contamination of the cDNA library.

### 2.5. miRNA Alignment

After sequence cleaning, reads were mapped to known miRNA and precursors of miRNAs (pre-miRNA) with miRanalyzer v 0.3 using the dataset of human miRNAs contained in miRBase [27]. Filtered files were converted to “read-count” format with groupReads.pl script built-in miRanalyzer standalone version. This function removes reads with less than 5 hits and also trims sequences up to 30 bp. miRanalyzer is a java tool that integrates short read alignment to genome and miRNome of reference using Bowtie aligner, allowing the introduction of external libraries or databases as a reference [28]. This method only considers those read counts which just map to one and no other sequence of the reference sequence. Annotation was carried out sequentially, first, read counts were aligned to known miRNAs and removed from the database after matching. Remaining reads that did not align with the miRNA database were mapped against pre-miRNA reference sequences of miRBase.

### 2.6. Differential Expression Analysis

The software package used to analyze differential expression among miRNA from distinct groups was DESeq2 [29], which works in R environment. This analytic tool is based on a negative binomial distribution model and infers probabilities before (*p*-value) and after False Discovery Rate correction (*q*-value), to spot differentially expressed miRNAs among groups of individuals. The variation in expression level is provided as log_2_ of fold change (FC). Dual comparisons were carried out between the aforementioned groups to assess the differences in expression levels of previously mapped miRNAs and pre-miRNAs. This approximation allows the evaluation of changes in miRNA expression due to the viremia level examining typical progressors before and after treatment, miRNA expression variations associated with LTNP phenotype compared to TP, and the miRNA profile linked to viremia control examining EC-LTNP versus vLTNP. Those miRNA with statistically significant differences in their expression are defined as differentially expressed (DE). R packages pheatmap, factoExtra, gridExtra, and ggplot2 were used for PCA and heatmaps analysis.

### 2.7. Target Genes Identification and Functional Analysis

Target genes of DE miRNAs were predicted and those that might be involved in HIV infection immunopathogenesis were further analyzed. First, miRNAs were linked to targets related to HIV infection through the information available in scientific literature, and subsequently, the predicted union sites and type of union in 3′-UTR were checked in TargetScanHuman v 7.1 [30]. 

Likely modifications in signaling pathways were assessed via miRNet, which allows making miRNA target identification and refinement, network visual exploration, and enrichment of altered pathways. We selected the DE miRNAs with the most robust statistical significance (LTNP vs. TP comparison), and we studied which genes were targeted specifically in two databases. The first one is composed of 212 genes included within Kyoto Enciclopedia of Genes and Genomes (KEGG) Human Immunodeficiency Virus 1 Interaction—*Homo sapiens* (human) pathway (hsa05170) [31]. Once the interacting genes with DE miRNA in this list were identified, we applied a filter within the HIV-1 infection KEGG route, highlighting those pathways which could be altered in LTNP. Alternatively, we used the 4668 genes retrieved from the HIV-1 host proteins interactome database available at NCBI [32,33,34,35,36] to filter the interacting genes with the DE miRNAs identified in LTNPs when compared to TPs. Finally, we conducted a pathway enrichment analysis within both networks using an empirical sampling approximation [37].

## 3. Results

### 3.1. Patient Characteristics

Patient’s records were provided by the LTNP-RIS and CoRIS cohorts alongside the classification of the samples—all the information is available in Table 1. Positive-HIV patients are considered LTNP when their CD4+ T lymphocyte counts are above 500 cells/mm^3^ for at least 10 years, in the absence of ART. This group is divided into two subgroups (*n* = 8) according to their capacity to control viral replication: elite controllers LTNP (EC-LTNP), defined as those with undetectable viremia or viral load < 2000 copies of viral RNA/mL in less than 25% of determinations during the follow-up, and viremic LTNP (vLTNP) that includes the other individuals with a viral load below 10,000 copies/mL in all the determinations during the follow-up. The whole set of LTNP was followed up to 10 years and diagnoses were registered between 1988 and 1999. Typical progression was defined as a loss of 50–100 CD4+ T cells/mm^3^ per year with a presence of >5000 copies of viral RNA/mL. After two years of ART, viral load declined below the detection level (<20 copies/mL). There are no noteworthy differences in age, gender, or provenance between the established groups. 

### 3.2. Next-Generation Sequencing Quality Assessment

A total amount of 393,350,988 reads were obtained in sequencing, with an average of 13,211,670 reads per patient. After adaptor trimming and data filtration as referred to in methods, the number of reads available for mapping reached 73.44% of initial reads (9,703,085 reads per patient). All sequences had an average Phred score per base above 38 after filtering. There were no statistically significant differences between the remaining numbers of reads between the phenotypical groups. 

### 3.3. miRNA Mapping

**Figure 1:**
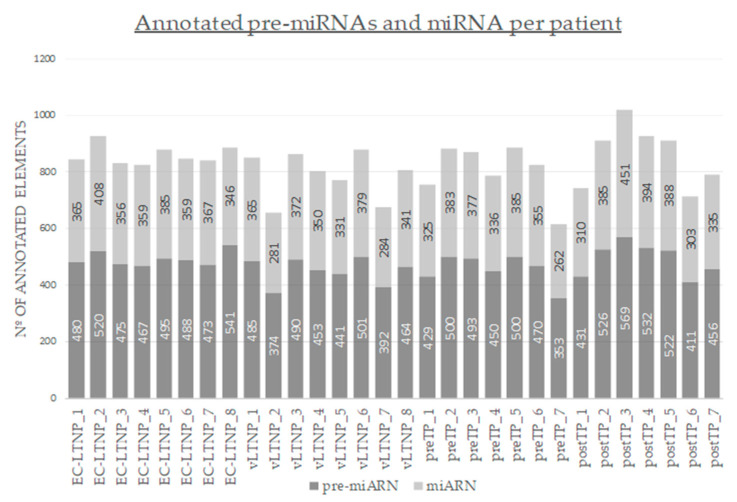
Number of identified mature miRNAs and precursor miRNAs (pre-miRNA) per patient.

Filtered sequences were mapped against different databases to obtain annotation of the reads. An average of 4,499,757 reads was mapped to known miRNAs and 4,692,188 reads were annotated as pre-miRNAs per patient. PBMC miRNome is formed by a mean (±SD) of 355 (±39) miRNAs and 472 (±48) pre-miRNAs (Figure 1). The hsa-miR-26a was identified as an overrepresented sequence in the whole set of patients, accounting up to a mean (SD) of 12.57% (±0.035%) of the aligned reads to miRNAs.

### 3.4. Global Comparison

**Figure 2:**
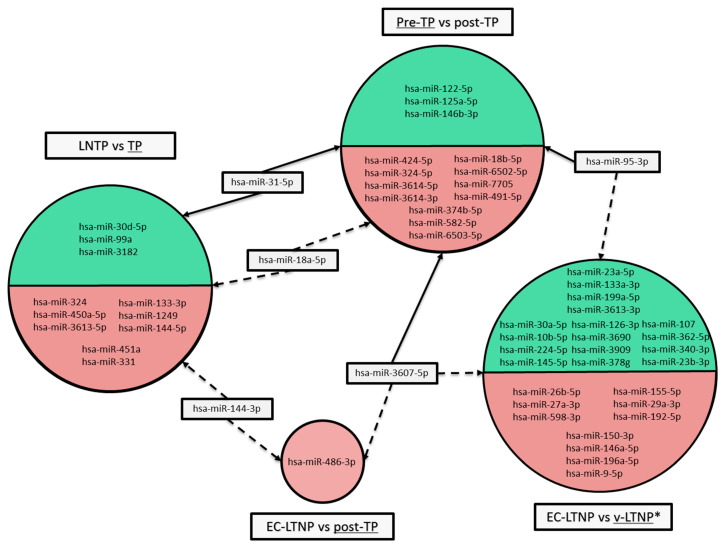
Summary of differentially expressed miRNAs (*q*-value < 0.1). Each comparison is represented as a circle split in two colors, green for upregulated miRNAs and red for downregulated ones. Overlapping miRNAs are depicted inside grey boxes. Solid lines illustrate upregulation in the corresponding group, while dotted lines show downregulation. Underlined phenotype indicates the reference group in each comparison. *Statistical significance of DE miRNA *p*-value < 0.05. LTNP (Long-term non-progressor); TP (Typical progressor); EC-LTNP (Elite controller Long-term non-progressor).

In order to describe miRNA expression alteration, study groups were compared in pairs. The analysis of differential expression confirms the modification of miRNA expression patterns related to distinct phenotypes. Figure 2 shows the lists of DE miRNAs generated in each comparison.

**Figure 3:**
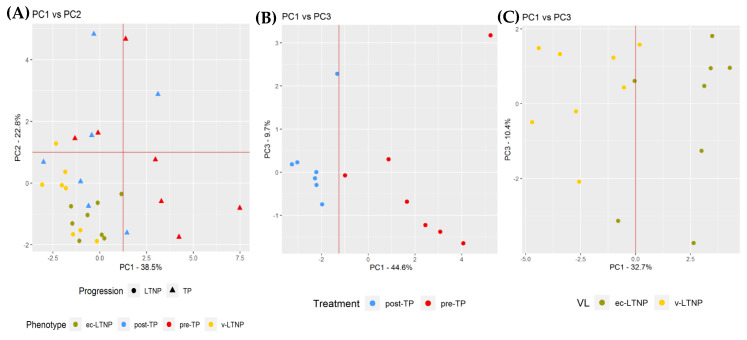
PCA analyses were carried out to identify relationships between distinctive miRNA expression patterns and rate of progression or viral load. DE miRNAs determined in each comparison were selected to generate the graphs (**A**) LTNP vs. TP, (**B**) pre-TP vs. post-TP, and (**C**) vLTNP vs. EC-LTNP. Supplementary Figures S1–S3 show the contribution and loadings of each miRNA in PCs. (**A**) All LTNP patients group together in negative values of PC1 and PC2. TP patients do not group attending to any PC values. (**B**) TP individuals are only separated by PC1. Patients receiving ART (blue) show lower values whereas its value varies before treatment (red). (**C**) EC-LTNPs present high scores on PC1 except for two patients which are close to vLNTPs scores.

Principal component analysis (PCA) was carried out so that the main miRNAs associated with each phenotype could be outlined (see Figure 3). Each graph was generated by selecting the DE miRNAs found in each comparison, in order to avoid background noise. At the representation of the whole group of samples, LTNPs cluster all together (Figure 3A). The most remarkable finding is that 80% of LTNPs present a negative value for PC1 and only hsa-miR-3182 contributes with a negative loading value. Coefficients from hsa-miR-18a-5p, hsa-3613-5p, and hsa-miR-324-5p contribute positively on this component. At PC2, hsa-miR30d-5p, hsa-miR-3182, and hsa-miR324-5p play a role as negative coefficients while hsa-miR451a, hsa-miR-144-3p, and hsa-miR-144-5p are the most relevant positive coefficients. Performing PCA in separate groups reveals discrimination of phenotypes according to ART administration, Figure 3B, and viral load control in LTNP individuals (Figure 3C). 

### 3.5. Differences in miRNA Expression Associated with Viral Replication

**Figure 4:**
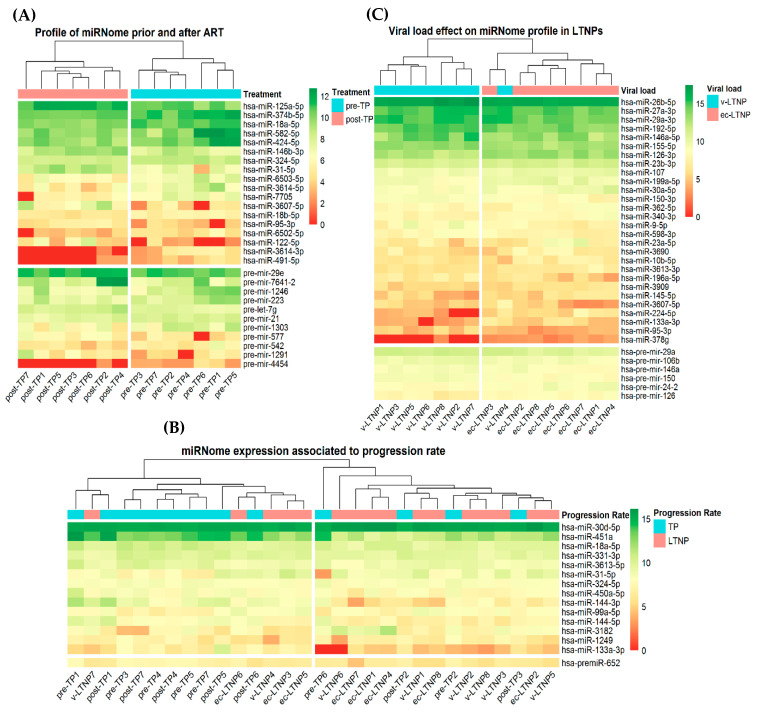
Heatmaps of differentially expressed miRNA and pre-miRNA for each comparison. Expression levels are represented as log_2_ of normalized counts per patient. Individuals were clustered using an unsupervised Euclidean approximation based on miRNA and pre-miRNA counts. The color scale on the right indicates the relative expression level of each miRNA, green being the higher values and red being the lower scores. (**A**) pre-TP vs. post-TP. Patients are split into their corresponding group accurately, showing that this set of miRNA and pre-miRNA is associated with the administration of treatment. (**B**) LTNP vs. TP. LTNP individuals are differentiated from TP, finding five mismatches. This panel of expression allocates correctly 71% of TP and 68.5% of LTNP. (**C**) EC-LTNP vs. v-LTNP. vLTNPs are associated together except for one patient who clusters with EC-LTNP.

Attending to paired comparisons individually, we first assessed TP groups in order to register miRNA expression profile alterations due to the presence of viremia. By comparing typical progressors before ART administration (pre-TP) and the same patients after ART (post-TP), 18 miRNA (*q*-value < 0.1), and 11 pre-miRNA (*p*-value < 0.05) were found to be dysregulated. Among them, hsa-miR-3614-5p (FC: −1.726), hsa-miR-3614-3p (FC: −2.618), hsa-miR-18a-5p (FC: −0.782), and hsa-miR-18b-5p (FC: −0.729) are downregulated after treatment, and miRNAs hsa-miR-3607-5p (FC: 2.050) and hsa-miR-31-5p (FC: 1.597) display upregulation. Although statistical significance after FDR is not reached for pre-miRNAs, hsa-pre-miR-1303 and hsa-pre-miR-4454 suffer a reduction in post-ART patients, with FC of (−1.481) and (−2.360) respectively. A complete list of DE miRNAs and pre-miRNAs can be consulted in Supplementary Table S1. In Figure 4A, we observe that pre-TP patients are highly differentiated from post-TP patients based on the expression level of the whole set of DE miRNA and pre-miRNA. Some of those miRNAs are present in other comparisons (Figure 2).

Table 2 presents a full list of experimentally validated targeted genes implicated in the HIV replication cycle, predicted union site, and type of union based on TargetScan v 7.0 analysis. 

### 3.6. Differences in miRNA Expression Associated with Disease Progression

In order to unravel the common miRNA expression pattern of LTNPs, miRNA profiles of the whole group of LTNPs were compared to those of TPs. Interestingly, 14 miRNAs and one pre-miRNA are differentially expressed (*q*-value < 0.1, Supplementary Table S2, Figure 4B) between these two groups. Some of them were downregulated in LTNPs, including hsa-miR-144-5p (FC: −1.013), hsa-miR-144-3p (FC: −1.086), hsa-miR-451a (FC: −1.276), and hsa-miR-18a-5p (FC: −0.644) while hsa-miR-30d-5p (FC: 0.315) was upregulated within this group. Regarding pre-miRNA, hsa-pre-miR-652 was downregulated (FC: −0.808) in LTNPs. Regulated genes involved in HIV infection by these miRNAs, such as *RAB14* or *DICER1,* are shown in Table 2.

### 3.7. Differences of miRNA Expression Associated with EC-LTNP Phenotype

In an effort to solve whether the post-transcriptional gene expression regulation by miRNA is accountable for the control of viral load in elite controllers, we performed a comparison between EC-LTNP and vLTNP miRNomes. The analysis showed a difference of 28 miRNAs and six pre-miRNAs (*p*-value < 0.05, Supplementary Table S3) between miRNomes of both groups (Figure 4C). Some targeted genes implicated in the viral cycle are presented in Table 2. 

Additionally, EC-LTNP was also compared to post-TP. There was found to be a difference in the expression level of three miRNAs (*q*-value < 0.1) and four pre-miRNAs (*p*-value < 0.05) between elite controllers and patients after treatment (Table 3). Peculiarly, hsa-miR-144-3p and hsa-miR-486-3p are detected both as DE miRNA and pre-miRNA, though only the mature form reached a high enough q-value to be statistically significant. In addition, hsa-miR-144 is detected as downregulated pre-miRNA in EC-LTNP when compared to vLTNP. Both in EC-LTNP vs vLTNP, and EC-LTNP vs post-TP comparisons, hsa-miR-3607-5p is a markedly downregulated miRNA in EC-LTNP, but this miRNA is upregulated in post-TPs when contrasted with the pre-TP group. 

### 3.8. Potentially Altered Pathways Regulated by DE miRNA Associated with Disease Progression

Subsequently, differentially expressed miRNAs between LTNP and TP were used in miRNet v.2.0 in order to elucidate the pathways that could be altered by these expression changes affecting disease progression. Two alternative approaches were used as reported in methods.

#### 3.8.1. DE miRNA Predictably Targets Various Genes Involved in the KEGG HIV-1 Infection Pathway

To explore the interactions of DE miRNAs and genes associated exclusively with the HIV infection cycle, we depicted the relationships of selected miRNAs with host proteins comprised in the KEGG HIV pathway. A total of 36 genes from the 212 genes participating in the KEGG route are regulated by 12 out of 14 differentially expressed miRNAs. A list of the targeted genes can be accessed in Supplementary Table S4.

**Figure 5:**
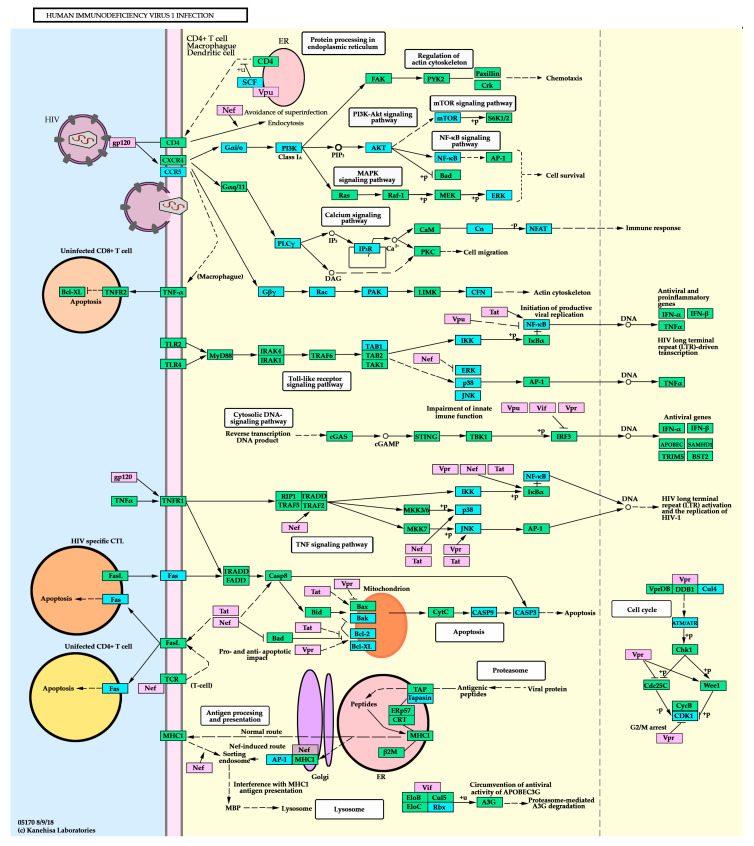
Human Immunodeficiency Virus 1 infection KEGG route. Mauve boxes represent viral genes while green and blue boxes show host genes. Blue boxes indicate targeted genes by DE miRNAs in LTNP compared to the TP group. White rounded rectangles reference to other KEGG maps. Circles: small molecules. Arrows: Molecular interaction or activation. Lines with perpendicular ends: inhibition. Dashed lines: Indirect effect or unknown relation. +p: phosphorylation. –p: dephosphorylation. +u: ubiquitination.

The main routes regulated by these DE miRNA involves functions related to cell survival (*FAS, AKT*), immune response, and viral replication (*TAB1, NF-κB, ITPR, PPP3CB, NFAT, CCR5),* or cytoskeleton reorganization and intracellular transport (*GBN5, RAC1, CFL1*). A whole map of interacting genes is shown in Figure 5. 

#### 3.8.2. Enrichment Analysis of Likely Altered Pathways Based on HIV-1 Human Interaction Database 

**Figure 6:**
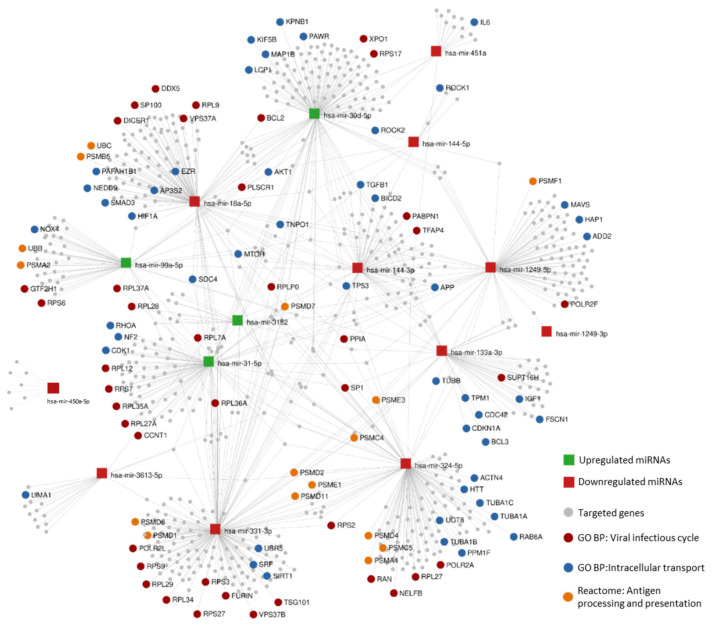
Interaction network of DE miRNAs and their targets included in the NCBI HIV-1 host genes interactome. Squares represent miRNAs and linked circles represent targeted genes. Red dots: Viral infectious cycle genes. Blue dots: Protein import into nucleus and translocation, Actin filament bundle assembly, and Cytoskeleton-dependent intracellular transport genes. Orange dots: Cross-presentation of soluble exogenous antigens (endosomes) and Antigen processing: Ubiquitination and Proteasome degradation genes. Viral infectious cycle shares *UBB* and *UBC* genes with antigen processing and presentation and *KPNB1, TGFB1,* and *MAVS* with cell transport.

A number of 789 genes are affected by the whole set of DE miRNAs found in LTNP versus TP (Figure 6). The enriched pathways analysis carried out in miRNet using this group of genes revealed that they are implicated in 13 KEGG pathways, 10 Gene Ontology Biological Processes, and 16 Reactome pathways. The list of pathways is presented in Supplementary Table S5. Phagosome (12 targeted genes), Leukocyte transendothelial migration (24 targeted genes) and Pathogenic *Escherichia coli* infection (11 targeted genes) are among the top five statistically significant KEGG routes, indicating a strong regulation of immune response in long-term non-progression. Cross-presentation of soluble exogenous antigens (endosomes), antigen processing, ubiquitination and proteasome degradation, or Vpu mediated degradation of CD4, could highlight a possible dysregulation in antigen presentation in LTNP attending to Reactome criteria. These three routes comprise a batch of 16 targeted genes in common. Moreover, there are pathways related to cell cycles as a regulation of apoptosis or a stabilization of p53 which may be altered by DE miRNAs, with a total of 18 regulated genes. Attending to the biological processes annotated in Gene Ontology, there are two main categories of great interest: cell transport, and viral infectious cycle. Cell transport consists of protein import into the nucleus, actin filament bundle assembly, and cytoskeleton-dependent intracellular transport, with a total of 48 genes controlled by DE miRNAs. The virus relies on these routes for the transport of viral products, from the entrance to the release of virions. The viral infectious cycle route, which accounts for 46 altered genes on this network, indicates a differential regulation of common host genes implicated in viral infection in long-term non-progressors. 

## 4. Discussion

In this study, we have assessed the miRNome of PBMCs from different phenotypes of HIV-infected people, which serves a purpose on screening crucial miRNA controlling gene expression in the context of HIV control and delayed progression. Despite this objective having been previously addressed by other research groups [63,64,65,66], no one has ever explored this issue through next-generation sequencing. This methodology offers many valuable advantages since it avoids design bias, cross-hybridization, and provides a wider dynamic range, more accuracy, and sensitivity [67]. Additionally, we have explored potentially regulated genes by DE miRNA relevant in the HIV cycle using an alternative approach that fuses network visualization and the HIV–host gene interaction database [36]. 

It is well established that viral infection has an impact on miRNA expression [68]. RNA interference is an ancient mechanism that plays a basic role in cellular functions as differentiation, cell cycle, and an innate antiviral defense. Hence, it was predictable that HIV developed weapons to counteract miRNA action as a survival strategy. Triboulet et al. [47] demonstrated that downregulation of Drosha and DGCR8, essential molecules in the putative miRNA synthesis process, enhanced viral production in vitro.

According to that, there are several examples of how HIV withholds miRNA synthesis machinery through a range of viral molecules known as RNA silencing suppressors (RSS) [19]. It has been proposed that the HIV Tat protein decreases miRNA expression due to the inhibition of Dicer machinery [69]. Our data support this tendency since samples from patients with an active viral replication (vLTNP and pre-TP) showed less total miRNAs/pre-miRNAs compared to EC-LTNP and post-TP individuals. Viral protein R (Vpr) may also act as an RSS, facilitating HIV infection of macrophages through Dicer ubiquitination and subsequent proteasomal degradation [70].

The existence of viral opposing forces to RNAi brings to light the relevance of miRNA in the host’s fight against the virus. Though its role in HIV control may be secondary and direct control of HIV mRNA could be easily overcome by 3′ UTR sequence mutation, miRNA exerts a fundamental role in the fine-tuning of inflammatory responses [71]. The dependence of HIV on host genes that participate in replication, such as NF-kB, explains the narrow relationship between miRNA shifts and HIV success. Thus, changes in the expression of host genes could be assisted by miRNAs, generating a favorable landscape for HIV control. In this paper, we have demonstrated a singular signature of miRNA expression associated with control of viremia and the rate of progression. Particularly, comparing miRNA profiles in patients before and after receiving ART, we have identified 18 miRNAs and 11 pre-miRNAs differentially expressed. Some of them have also been described by other authors [63,72] as hsa-miR-31-5p, strongly upregulated in patients under ART in our study or hsa-miR-18a-5p, downregulated after ART. The findings of Zhang et al. [72] correlate the higher expression of hsa-miR -31-5p to T cell protection against spontaneous and activation-induced cell death. Moreover, this miRNA was correlated with low viremia and high CD4+ T cell counts being significantly upregulated in elite controllers when compared to chronically infected HIV patients. These data support our results, showing an increase in hsa-miR-31-5p levels in typical progressors on ART. Respecting hsa-miR-18a-5p, it was described in [66] to be positively correlated to CD4+ T cell counts, although in this study we observed a lower level of expression in post-TP patients. It has been demonstrated that Vpr activation of HIV-1 LTR is dependent on HIF-1α, which is induced by Vpr activation of the oxidative stress pathway [41]. Typical progressors overexpress miR-18a-5p before ART administration in our study, which could be a post-transcriptional fine-tuning mechanism to counteract HIF-1α enhanced expression produced by Vpr action.

After analyzing miRNA profile changes affected by high levels of viremia, we explored which miRNAs could be playing a role in long-term non-progression. We detected 4 miRNAs upregulated and 10 miRNAs and 1 pre-miRNA downregulated in LTNPs compared to typical progressors. In an attempt to stress those miRNAs most related to the LTNP phenotype, we carried out a PCA analysis. This analysis pointed out that low levels of miR-451a, miR-18a-5p, miR-324-5p, and miR-144-3p mostly separated LTNP from typical progressors. Remarkably, miR-18a-5p is also downregulated in LTNP as it was in patients after receiving ART. TargetScan prediction analysis and in vitro assays showed that Dicer is one of the predicted targets of miR-18a-5p [73]. Taking into account that HIV withholds Dicer pre-miR processing activity, downregulating miR-18a-5p could be a host strategy to increment Dicer translation, overcoming RSS activity. Besides, miR-451a and miR-144 regulate RAB14 expression [44], a key molecule in enveloping glycoprotein complex incorporation during HIV-1 virion maturation [45]. Both miRNAs are expressed from the same precursor codified in chromosome 17, despite final steps in miRNA processing differing between them [74]. Dicer participates in miR-144 maturation while miR-451a does not follow the canonical course, being directly recognized by AGO2, suggesting that host cells use a wide range of mechanisms to bypass virus strategies of RNAi silencing. Therefore, downregulation of miR-18a-5p, miR-451a, and miR-144 in LTNP are associated with Dicer modulation and virion assembly. Further work must be conducted in order to shed a light on the potential role of miR-18a regulating Dicer expression in the context of HIV infection. Another interesting phenotype in HIV infection resistance is HIV-exposed seronegative individuals (HESN), highly virus-exposed individuals persistently seronegative. The expression profile of 84 miRNA of these patients has been assessed in unstimulated PBMC of HESN, HIV+ individuals, and healthy controls [75]. Interestingly, miR-99a is also upregulated in HESN as we reported in our LTNP. This miRNA targets AKT and mTOR, central components of the mTORC1 complex [76]. A double suppression of the mTOR pathway helps in the early stage of HIV infection, blocking the entry to the cell through CCR5 recycling and interfering with induced and basal transcription [77]. Although only one DE miRNA is regulated similarly in HESN compared to our LTNP patients, it should be noticed that resistance mechanisms in HESN may differ from those of LTNPs since the latter regulates disease progression and HESN is probably involved in HIV-1 acquisition.

Elite controllers represent the best natural scenario controlling HIV infection. Mimicking their biological status may turn into a successful immunotherapy to achieve a functional cure. Hence, we investigated the possibility of finding some miRNA(s) responsible for HIV control in the context of non-progression. Despite that we did not find significant DE miRNA after FDR correction between EC-LTNP and vLTNP, we could identify 28 miRNAs and six pre-miRNAs in this comparison (*p*-value < 0.05 significance). Although a relaxed threshold for significance was applied, several DE miRNAs found in our study have also been reported in other studies, indicating that our threshold may be affected by the number of samples. For instance, miR-29a has been extensively studied, though it is difficult to discern if its downregulation in EC is a cause or a consequence of low viral replication level. Low levels of miR-29 have been registered in EC-LTNP compared to vLNTP via microarrays [64]. Some studies depict miR-29a as an anti-HIV miRNA since it targets 3′UTR HIV mRNAs, and higher levels of this miRNA reduced viral production [78,79]. Therefore, PBMCs in vLTNP might be overexpressing miR-29a to hamper Nef expression, this being upregulated as a consequence of higher virus titer. However, HIV mRNA secondary structures handicap miRNA interaction and Tat may directly downregulate miR-29a expression via NF-kB [80]. Diversely, miR-29b (analog to miR-29a) has also a regulatory role on CCNT1, an important co-factor of Tat [81]. The activation of CD4+ T cells upregulates CCTN1 levels independently of mRNA levels, suggesting an RNAi mechanism in charge of this change. Chiang et al. [82] described that miR-29a may indirectly regulate post-transcriptional expression of CCTN1, suggesting that low levels of miR-29 observed in EC may correspond with higher activation of CD4+ T cells. Whether this over activation of a specific CD4+ T cell pool could play a role in controlling virus is yet to be determined. Another interesting deregulated miRNA is miR-155-5p. This miRNA was downregulated in LTNP compared to rapid and typical progressors and is proposed as a critical setting for long-term non-progression [83]. In fact, Witwer et al. [63] described a lower amount of miR-155 in PBMCs from healthy donors compared to viremic HIV-infected patients and miR-155 levels from elite controllers in our study are similar to those of healthy donors. ADAM10 and TPNO3 have been suggested as targets of this miRNA, and their part in nuclear transport of the preintegration complex is of the essence for the establishment of proviral DNA [84]. Rodriguez-Mora et al. showed that a defective mutation in TPNO3 confers protection against HIV infection [85]. This fact bolsters the relevance of this miRNA in the control of the virus and further research is necessary.

Finally, we detected three DE miRNA in EC-LTNP when compared to post-TP, reducing the impact of the viremia effect in miRNA expression. We found that miR-144-3p, miR-486-3p, and miR-3607-5p were differentially expressed. We also found miR-144-3p downregulated in LTNP, comparing them to TP and it is noteworthy to mention its level of expression is also lower in EC-LTNP than in vLTNP. Besides, Duskova et al. showed that miR-144-3p is downregulated in healthy patients compared to HIV-infected individuals with high viral loads. Moreover, miR-144 possibly targets IL-6, which emphasizes the resemblance of EC-LTNP immunological status with healthy donors, suggesting an interesting performance on virus replication control [65]. Concerning miR-486-3p, it has been reported to be downregulated in HIV/HVC coinfected patients when compared to healthy donors [86] and in LTNPs in comparison to aviremic HIV-infected patients under ART [87]. However, the function of this miRNA has not been assayed in the HIV context. Attending to their predicted targets, miR-486-3p controls PTEN phosphatase expression (all together with miR-29a-3p). PTEN dephosphorylates AKT and FOXO3 and its activity is enhanced in the presence of Tat in T lymphocytes [88]. Memory CD4+ T cells from EC-LTNP show a high level of phosphorylation of FOXO3, which is related to increased viral persistence; therefore, PTEN should be inhibited in these cells [89]. In our EC-LTNP patients, miR-486-3p is downregulated and this would imply an enhancement of the expression of PTEN, therefore dephosphorylating FOXO3 and inducing apoptosis in CD4+ T cells, opposite to Grevenynghe data [89]. However, Kim et al. depict how HIV Tat inhibits PTEN in macrophages in order to increase their survival, meaning miR-486-3p upregulation in patients with a detectable viral load could be counteracting HIV Tat activity. This mechanism would not be required in EC-LTNP because of its undetectable viral load and thus, miR-486-3p is downregulated.

These results should be interpreted with caution owing to the constraints in our study. One downside regarding our methodology is the limited sample size given the extraordinary nature of EC-LTNPs’ phenotype, and this could influence the statistical significance reached in DE miRNA analysis. On the other hand, a higher percentage of HCV-coinfection was found in LTNP groups as compared to typical progressors due to the evolution of HIV epidemics in Spain. However, differences in miRNA expression were found between EC-LTNP and vLTNP groups despite their similar HCV co-infection levels. Although HCV-coinfection cannot be ruled out as a confounding factor in the analyses, the results between LTNP groups suggest that it does not represent an issue. Moreover, our investigation has only been focused on PBMC so far, hindering the assignment of a particular miRNA expression pattern to a specific cell population. Relative proportions of each cell population in the PBMC pool may vary among patients, but we consider PBMC miRNA profile characterization as a first step in describing the main differences between phenotypes. Alterations in the expression in a particular population of immune cells may be masked or weakened by the absence or reciprocal shifts in miRNA profiles of other sets, sometimes larger in number. These issues are reserved for future work. Although it is generally assumed that miRNA expression levels are directly correlated with an effect caused in cells, this might be an oversimplification and has not been demonstrated across various miRNA and their targets [84,90]. Thus, we have analyzed all DE miRNA independently of their level of expression and we encourage validation of their role in HIV infection through in vitro assays. This effect may have been produced because of the lower number of samples. Be that as it may, it is interesting to highlight these miRNAs because of their potential role in viral replication control. Some of the differentially expressed miRNAs have been also detected as deregulated in other comparisons, such as hsa-miR-3607-5p (FC: −0.766) and hsa-miR-29a-3p (FC: −0.529) being downregulated in EC vs. vLTNP and upregulated in post-TP vs. TP. 

To summarize, this paper is the first report on PBMCs miRNome from HIV-1 infected individuals with different progression and virus control ability through NGS technology. Profiling with alternative methods generates complementary data to other research works, so describing PBMC miRNome by NGS not only reinforces previous miRNA results detected in other platforms but also may cover a distinct set of miRNAs, thereby letting us see the whole picture. We have exposed expression changes in the miRNome of patients with a typical progression pattern after receiving ART. In addition, we described the miRNA signature of LTNP and EC-LTNP. We propose that combined action of various miRNAs like miR-18a-5p, miR-29a-3p, miR-155-5p, miR-144-3p, miR-486-3p, and miR-3607-5p orchestrate host and/or viral gene expression and this leads to a reduced progression in AIDS and/or HIV control. 

## 5. Conclusions

Our results show miRNA expression levels are associated with viral load context and rate of disease progression. The main discovery of this work is the description of DE miRNA associated with LTNP and EC-LTNP. Robust downregulation of miR-144-3p, miR-451a, miR-18a-5p, and miR-324 is observed in LTNP compared to TP. This set of miRNA is associated with potential changes in the regulatory network of the immune response (leukocyte migration, antigen processing, and cross-presentation) and viral processes like cell transport or viral entry. EC-LTNP miRNomes differ modestly from that of vLTNP but when compared to ART-treated TP, they exhibit a strong downregulation of miR-486-3p and miR-144-3p, mirroring the levels of healthy individuals. Moreover, the administration of ART provokes a change in miRNA expression profiles in PBMC like the upregulation in miR-31-5p and miR-18a-5p, associated with the control of genes of the inflammatory response as HIF-1a, relevant for HIV replication. This shift is related to the reduction of viral load in ART patients.

## Figures and Tables

**Table 1:** Clinical parameters of the patients included in the analysis.

Group	Patient ID	Sex	Origin	CD4+ T Cell Count (Cell/mm^3^)	Via of Infection	HCV Co-Infection
EC-LTNP	EC-LTNP-1	M	European	635	IDU	Yes
	EC-LTNP-2	M	European	555	IDU	Yes
	EC-LTNP-3	F	European	596	IDU	Yes
	EC-LTNP-4	F	European	1081	SEX	No
	EC-LTNP-5	F	European	603	SEX	Yes
	EC-LTNP-6	M	European	640	IDU	Yes
	EC-LTNP-7	M	European	514	IDU	Yes
	EC-LTNP-8	M	European	708	IDU	Yes
vLTNP	vLTNP-1	M	European	625	SEX	No
	vLTNP-2	M	European	590	IDU	Yes
	vLTNP-3	M	European	1049	IDU	Yes
	vLTNP-4	M	European	593	IDU	Yes
	vLTNP-5	F	European	492	IDU	Yes
	vLTNP-6	M	European	NA	SEX	No
	vLTNP-7	M	European	889	IDU	No
	vLTNP-8	M	European	929	IDU	Yes
pre-TP	pre-TP-1	M	European	290	IDU	Yes
	pre-TP-2	M	European	332	SEX	No
	pre-TP-3	M	European	397	SEX	No
	pre-TP-4	F	European	270	SEX	No
	pre-TP-5	M	European	624	SEX	No
	pre-TP-6	M	European	157	SEX	No
	pre-TP-7	M	European	47	SEX	No
post-TP	post-TP-1	M	European	720	IDU	Yes
	post-TP-2	M	European	525	SEX	No
	post-TP-3	M	European	550	SEX	No
	post-TP-4	F	European	650	SEX	No
	post-TP-5	M	European	439	SEX	No
	post-TP-6	M	European	415	SEX	No
	post-TP-7	M	European	578	SEX	No

M: Male; F: Female; UDI: Intravenous Drug User; SEX: Sexual transmission.

**Table 2:** List of genes involved in AIDS immunopathogenesis targeted by DE miRNAs.

Group	Gene	miRNA	Expression Change	3′ UTR Predicted Site	Canonical Site Types ^1^	References ^2^
pre-TP vs. post-TP	*SOCS2*	miR-424-5p	↓	2253–2259	7mer-1A	[38,39]
	*HIF1A*	miR-18b-5p	↓	409–415	7mer-m8	[40,41]
	*AKT1*	miR-374b-5p	↓	2434–2440	7mer-1A	[42,43]
	*WNT16*	miR-374b-5p	↓	1262–1269	8mer	
LTNP vs. TP	*RAB14*	miR-144-5p	↓	361–367	7mer-m8	[44,45]
		miR-144-3p	↓	1052–1058	7mer-1A	
		miR-451a	↓	3207–3213	7mer-m8	
	*DICER1*	miR-18a-5p	↓	1201–1208	7mer-m8	[46,47]
			↓	4300–4307	8mer	
	*ING5*	miR-331-3p	↓	4147–4154	8mer	[48,49,50]
	*MTORC1*	miR-99a-5p	↑	295–301	7mer-m8	[51,52]
	*DNMT3*	miR-450a-5p	↓	649–655	7mer-1A	[53,54,55]
EC-LTNP vs. vLTNP	*TRAF6*	miR-146a-5p	↓	40–47	8mer	[56,57,58]
	*IRAK1*			473–480	8mer	
	*PTEN*	miR-106^3^	↓	272–278	7mer-m8	[59,60]
	*IL21R*	miR-30a-5p	↑	125–132	8mer	[61,62]

An underlined phenotype indicates the reference group in differential expression analysis. ^1^ Site types: *8mer*, an exact match to positions 2–8 of the miRNA followed by an ‘A’; *7mer-m8*, an exact match to positions 2–8 of the miRNA; *7mer-A1*, an exact match to positions 2–7 of the miRNA followed by an ‘A. ^2^ References to experimental evidence of gene expression regulation by miRNAs and interaction of these proteins in HIV infection are provided. ^3^ Found as DE pre-miRNA in this comparison.

**Table 3:** Differentially expressed miRNA between EC-LTNP and post-TP.

miRNA	log_2_(FC)	*p*-Value	*q*-Value
miR-3607-5p	−1.6073	0.00001	0.0064
miR-486-3p	−1.0910	0.00002	0.0071
miR-144-3p	−1.2943	0.0003	0.0892

The reference group in this comparison is post-TP.

## Supplementary Materials

The following are available online at https://www.mdpi.com/2077-0383/9/8/2452/s1, **Figure S1:** Loading values and contribution per variable in PCA analysis a) LTNP vs. TP, **Figure S2:** Loading values and contribution per variable in PCA analysis b) pre-TP and post-TP, **Figure S3:** Loading values and contribution per variable in PCA analysis c) EC-LTNP vs. vLTNP, **Table S1:** List of DE miRNA and pre-miRNA between pre-TP and post-TP, **Table S2:** List of DE miRNA and pre-miRNA between LTNP and TP, **Table S3:** List of DE miRNA and pre-miRNA between EC-LTNP and vLTNP, **Table S4:** List of targeted genes participating in KEGG routes within KEGG Human Immunodeficiency Virus 1 Infection, **Table S5:** List of KEGG pathways, Gene Ontology: Biological Processes and Reactome enriched with altered genes associated with disease progression.
