# Supplementary material for: Insight in miRNome of Long-Term Non-Progressors and Elite Controllers Exposes Potential RNAi Role in Restraining HIV-1 Infection
Source: J Clin Med. 2020 Jul 31;9(8):2452. doi: 10.3390/jcm9082452 (PMC7464121; doi:10.3390/jcm9082452)
Supplement: Supplementary file 1 [file jcm-09-02452-s001.pdf]

Journal of
Clinical Medicine

SUPPLEMENTARY MATERIAL

Supplementary Materials: The following are available online at www.mdpi.com/xxx/s1

Figure S1: Loading values and contribution per variable in PCA analysis a) LTNP vs TP.

Figure S2: Loading values and contribution per variable in PCA analysis b) pre-TP and post-TP.

Figure S3: Loading values and contribution per variable in PCA analysis c) EC-LTNP vs vLTNP.

Table S1: List of DE miRNA and pre-miRNA between pre-TP and post-TP

Table S2. List of DE miRNA and pre-miRNA between LTNP and TP.

Table S3. List of DE miRNA and pre-miRNA between EC-LTNP and vLTNP.

Table S4. List of targeted genes participating in KEGG routes within KEGG Human Immunodeficiency Virus 1 Infection.

Table S5: List of KEGG pathways, Gene Ontology: Biological Processes and Reactome enriched with altered genes associated with disease progression.

Loading values for PC in PCA (**A**)

miRNA	PC1	PC2
miR-451a	0.174	0.487
miR-144-5p	0.190	0.432
miR-18a-5p	0.380	-0.079
miR-144-3p	0.168	0.485
miR-133a-3p	0.212	0.144
miR-331-3p	0.368	-0.135
miR-3613-5p	0.377	-0.038
miR-99a-5p	0.083	-0.163
miR-30d-5p	0.240	-0.345
miR-3182	-0.025	-0.212
miR-450a-5p	0.330	0.097
miR-1249	0.350	-0.142
miR-324-5p	0.376	-0.193
miR-31-5p	0.018	-0.192

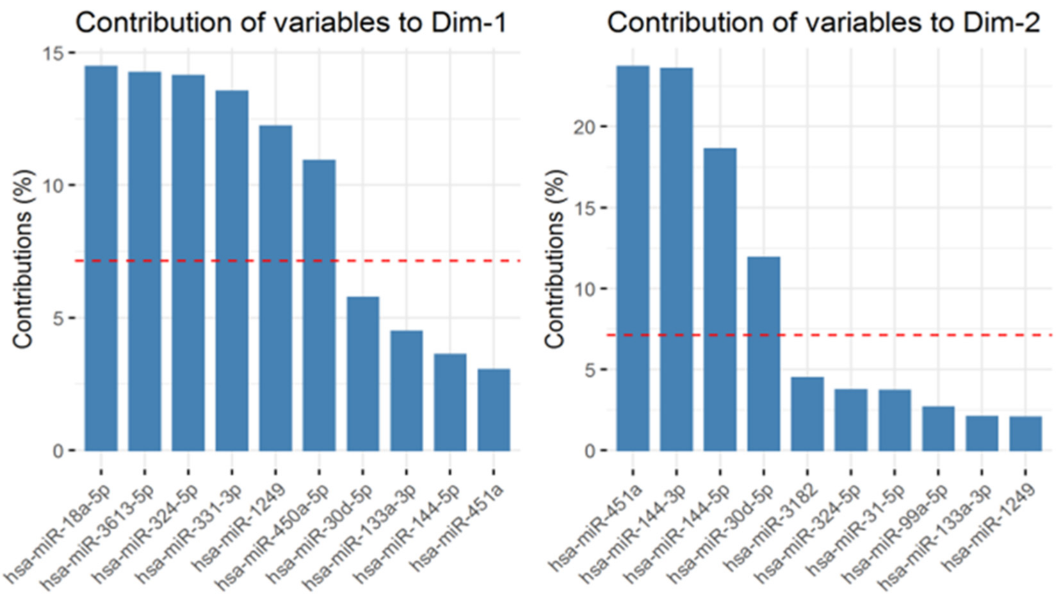

Figure S1. Loading values and contribution per variable in PCA analysis (**A**) LTNP vs TP.

Loading values for PC in PCA (**B**)

miRNA	PC1	PC3
miR-3614-3p	0.270	-0.196
miR-3607-5p	-0.093	0.372
miR-31-5p	-0.131	0.072
miR-3614-5p	0.248	-0.329
miR-7705	0.277	-0.139
miR-125a-5p	-0.093	-0.229
miR-18a-5p	0.271	-0.005
miR-122-5p	-0.163	0.029
miR-146b-3p	-0.048	-0.175
miR-18b-5p	0.278	-0.196
miR-374b-5p	0.269	0.372
miR-6502-5p	0.279	-0.283
miR-6503-3p	0.297	0.186
miR-95-3p	-0.115	0.257
miR-491-5p	0.323	0.088
miR-324-5p	0.265	-0.056
miR-582-5p	0.270	0.315
miR-424-5p	0.277	0.372

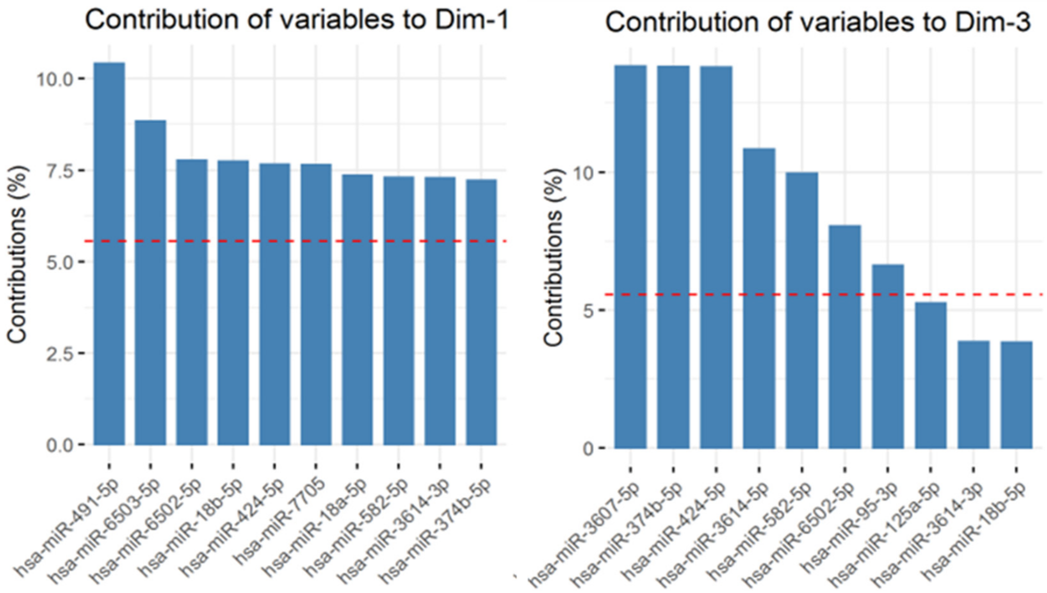

Figure S2. Loading values and contribution per variable in PCA analysis (**B**) pre-TP and post-TP.

Loading values for PC in PCA (**C**)

miRNA	PC1	PC3
miR-107	0.289	-0.012
miR-150-3p	-0.004	0.030
miR-155-5p	0.033	0.265
miR-23a-5p	0.202	-0.107
miR-30a-5p	0.229	-0.033
miR-9-5p	-0.115	-0.010
miR-126-3p	0.244	-0.246
miR-133a-3p	0.125	-0.452
miR-199a-5p	0.227	-0.234
miR-95-3p	-0.058	0.242
miR-10b-5p	0.237	0.135
miR-145-5p	0.283	-0.006
miR-196a-5p	-0.067	0.245
miR-224-5p	0.219	0.065
miR-3607-5p	-0.069	-0.256
miR-3909	0.275	0.012
miR-378g	0.254	-0.011
miR-26b-5p	0.114	0.176
miR-146a-5p	-0.046	0.209
miR-192-5p	-0.028	-0.074
miR-23b-3p	0.282	-0.014
miR-27a-3p	-0.013	-0.281
miR-29a-3p	-0.030	-0.203
miR-340-3p	0.277	0.124
miR-3613-3p	0.228	0.174
miR-362-5p	0.289	0.256
miR-3690	0.182	-0.120
miR-598-3p	-0.063	-0.251

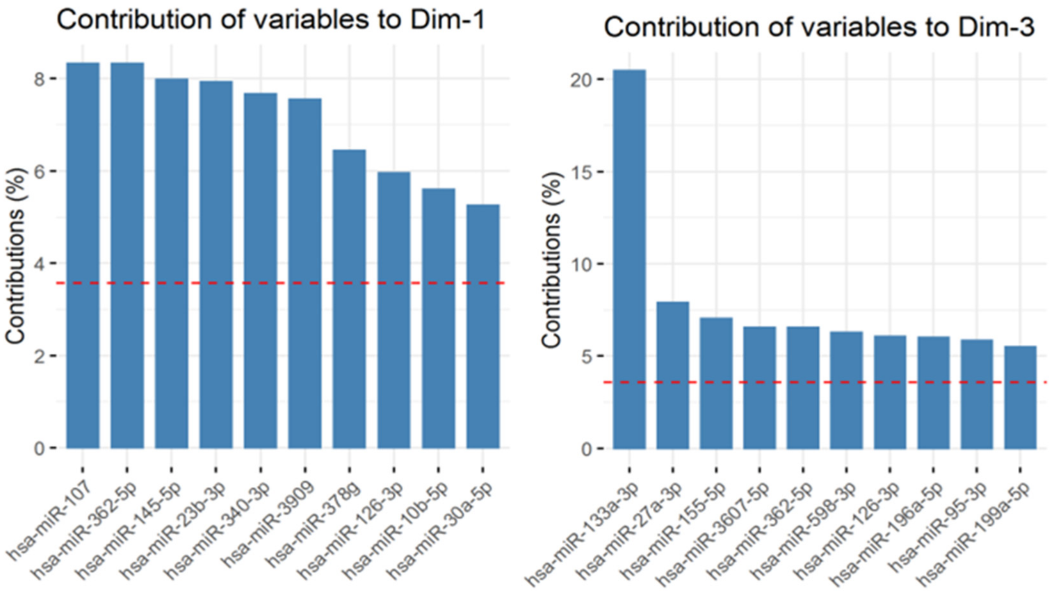

Figure S3. Loading values and contribution per variable in PCA analysis (**C**) EC-LTNP vs vLTNP.

Table S1. List of DE miRNA and pre-miRNA between pre-TP and post-TP.

miRNA	log_2(FC)	p-value	q-value
miR-3614-3p	-2.6184	0.0000	0.0001
miR-3607-5p	2.0502	0.0000	0.0055
miR-31-5p	1.5973	0.0000	0.0087
miR-3614-5p	-1.7258	0.0001	0.0124
miR-7705	-1.4787	0.0001	0.0124
miR-125a-5p	1.1152	0.0002	0.0148
miR-18a-5p	-0.7817	0.0004	0.0298
miR-122-5p	1.5869	0.0014	0.0892
miR-146b-3p	1.0427	0.0027	0.0916
miR-18b-5p	-0.7289	0.0019	0.0916
miR-374b-5p	-0.7749	0.0024	0.0916
miR-6502-5p	-1.1761	0.0021	0.0916
miR-6503-5p	-1.1607	0.0025	0.0916
miR-95-3p	1.2279	0.0026	0.0916
miR-491-5p	-1.5297	0.0021	0.0916
miR-324-5p	-0.4469	0.0033	0.0978
miR-582-5p	-1.2607	0.0031	0.0978
miR-424-5p	-1.1210	0.0036	0.0996
**pre- miRNA**	**log_2(FC)**	**p-value**	**p-adj**
miR-1303	-1.4809	0.0020	0.2131
miR-4454	-2.3603	0.0014	0.2131
let-7g	0.8160	0.0054	0.3807
miR-223	-1.0589	0.0114	0.4766
miR-577	1.2793	0.0096	0.4766
miR-7641-2	1.6784	0.0144	0.5048
miR-542	-1.0674	0.0213	0.6385
miR-29a	0.5119	0.0271	0.7109
miR-1290	-1.4544	0.0331	0.7391
miR-3607	1.1819	0.0352	0.7391
miR-1246	-1.1636	0.0428	0.8170

The reference group in this comparison is pre-TP.

Table S2. List of DE miRNA and pre-miRNA between LTNP and TP.

miRNA	log_2(FC)	p-value	q-value
miR-451a	-1.2761	0.0000	0.0056
miR-144-5p	-1.0131	0.0001	0.0116
miR-18a-5p	-0.6438	0.0001	0.0116
miR-144-3p	-1.0864	0.0003	0.0234
miR-133a-3p	-1.0433	0.0006	0.0283
miR-331-3p	-0.4770	0.0007	0.0283
miR-3613-5p	-0.6323	0.0007	0.0283
miR-99a-5p	0.6859	0.0008	0.0288
miR-30d-5p	0.3144	0.0013	0.0368
miR-3182	0.8843	0.0032	0.0836
miR-450a-5p	-0.6731	0.0036	0.0852
miR-1249	-0.6854	0.0041	0.0888
miR-324-5p	-0.3222	0.0047	0.0938
miR-31-5p	0.7606	0.0051	0.0954
**pre- miRNA**	**log_2(FC)**	**p-value**	**p-adj**
mir-652	-0.8082	0.00021	0.04426

The reference group in this comparison is TP.

Table S3. List of DE miRNA and pre-miRNA between EC-LTNP and vLTNP.

miRNA	log_2(FC)	p-value	q-value
miR-107	0.4492	0.0052	0.8045
miR-150-3p	-0.6068	0.0060	0.8045
miR-155-5p	-0.5216	0.0069	0.8045
miR-23a-5p	1.0287	0.0018	0.8045
miR-30a-5p	0.7411	0.0044	0.8045
miR-9-5p	-0.8923	0.0026	0.8045
miR-126-3p	0.6261	0.0088	0.8237
miR-133a-3p	0.8364	0.0118	0.8237
miR-199a-5p	0.6402	0.0108	0.8237
miR-95-3p	-0.7616	0.0111	0.8237
miR-10b-5p	0.6878	0.0170	0.8351
miR-145-5p	0.7437	0.0166	0.8351
miR-196a-5p	-0.7444	0.0164	0.8351
miR-224-5p	0.8078	0.0148	0.8351
miR-3607-5p	-0.7658	0.0202	0.8351
miR-3909	0.5199	0.0204	0.8351
miR-378g	0.7364	0.0196	0.8351
miR-26b-5p	-0.3338	0.0254	0.9833
miR-146a-5p	-0.6279	0.0464	0.9857
miR-192-5p	-0.5500	0.0310	0.9857
miR-23b-3p	0.3326	0.0485	0.9857
miR-27a-3p	-0.4641	0.0329	0.9857
miR-29a-3p	-0.5291	0.0314	0.9857
miR-340-3p	0.4431	0.0449	0.9857
miR-3613-3p	0.5118	0.0461	0.9857
miR-362-5p	0.4833	0.0394	0.9857
miR-3690	0.6223	0.0493	0.9857
miR-598-3p	-0.4861	0.0408	0.9857
**pre- miRNA**	**log_2(FC)**	**p-value**	**p-adj**
mir-29a	-0.6724	0.0013	0.2560
mir-126	0.7236	0.0039	0.3832
mir-150	-0.5860	0.0075	0.4839
mir-24-2	0.6354	0.0104	0.4996
mir-144	-1.1369	0.0483	0.7605
mir-106b	-0.4494	0.0457	0.9832

The reference group in this comparison is vLTNP.

Table S4. List of targeted genes participating in KEGG routes within KEGG Human Immunodeficiency Virus 1 Infection.

KEGG route	Targeted gene	miRNA	miRNA expression in LTNP vs TP
AKT	*AKT*	miR-99a-5p	↑
		miR-451a	↓
AP-1	*AP1G1*	miR-144-3p	↓
	AP1M1	miR-3182	↑
ATM/ATR	*ATM*	miR-18a-5p	↓
		miR-30d-5p	↑
Bak	*BAK1*	miR-331-3p	↓
Bcl-2	*BCL2*	miR-451a	↓
		miR-18a-5p	↓
Bcl-XL	*BCL2L1*	miR-1249	↓
		miR-133a-3p	↓
CASP3	*CASP3*	miR-30d-5p	↑
CASP9	*CASP9*	miR-133a-3p	↓
CCR5	*CCR5*	miR-3182	↑
CDK1	*CDK1*	miR-31-5p	↑
CFN	*CFL1*	miR-324-5p	↓
Cn	*PPP3CB*	miR-30d-5p	↑
Cul4	*CUL4A*	miR-18a-5p	↓
ERK	*MAPK1*	miR-451a	↓
Fas	*FAS*	miR-18a-5p	↓
Gαi/o	*GNAI2*	miR-30d-5p	↑
Gβγ	*GNB1*	miR-324-5p	↓
	GNB2	miR-324-5p	↓
		miR-331-3p	↓
	GNG12	miR-144-3p	↓
IKK	*IKBKB*	miR-451a	↓
IP3R	*ITPR2*	miR-324-5p	↓
JNK	*MAPK8*	miR-30d-5p	↑
mTOR	*mTOR*	miR-99a-5p	↑
		miR-144-3p	↓
NFAT	*NFATC1*	miR-324-5p	↓
NFKb	*RELA*	miR-324-5p	↓
p38	*MAPK13*	miR-18a-5p	↓
PAK	*PAK4*	miR-324-5p	↓
PI3K	*PI3K*	miR-30d-5p	↑
		miR-133a-3p	↓
	PIK3R3	miR-331-3p	↓
PLCy	*PLCG2*	miR-1249	↓
Rac	*RAC1*	miR-144-3p	↓
Rbx	*RNF7*	miR-331-3p	↓
SCF	*FBXW11*	miR-450a-5p	↓
TAB1	*TAB1*	miR-30d-5p	↑
Tapasin	*TAPBP*	miR-31-5p	↑

Table S5: List of KEGG pathways, Gene Ontology: Biological Processes and Reactome enriched with altered genes associated with disease progression.

KEGG pathways enriched in altered genes	Total	Expected	Hits	p-value
Phagosome	53	4.1	12	0.003
Cardiac muscle contraction	12	0.928	4	0.007
Leukocyte transendothelial migration	108	8.35	24	0.012
Pathogenic Escherichia coli infection	35	2.71	11	0.013
Asthma	7	0.541	1	0.014
Pentose phosphate pathway	27	2.09	6	0.017
Parkinson's disease	18	1.39	5	0.023
Pancreatic cancer	69	5.34	24	0.03
Autoimmune thyroid disease	10	0.774	2	0.03
Colorectal cancer	49	3.79	18	0.037
Amyotrophic lateral sclerosis (ALS)	39	3.02	9	0.038
Hypertrophic cardiomyopathy (HCM)	25	1.93	6	0.041
Type II diabetes mellitus	48	3.71	10	0.048
**GO:BP functions enriched in targeted genes**	**Total**	**Expected**	**Hits**	**p-value**
Protein import into nucleus, translocation	39	2.02	13	0.008
DNA damage response, signal transduction by p53 class mediator	117	6.07	28	0.01
Actin filament bundle assembly	83	4.3	20	0.014
Glucose catabolic process	117	6.07	17	0.018
Focal adhesion assembly	44	2.28	12	0.018
Signal transduction in response to DNA damage	129	6.69	29	0.02
Bone remodeling	63	3.27	11	0.038
Cytoskeleton-dependent intracellular transport	76	3.94	16	0.039
S phase of mitotic cell cycle	144	7.47	31	0.043
Viral infectious cycle	241	12.5	46	0.05
**Reactome pathways enriched in targeted genes**	**Total**	**Expected**	**Hits**	**p-value**
Cross-presentation of soluble exogenous antigens (endosomes)	47	3.49	14	0
Regulation of Apoptosis	50	3.71	16	0
Regulation of activated PAK-2p34 by proteasome mediated degradation	49	3.64	16	0
Autodegradation of the E3 ubiquitin ligase COP1	51	3.79	18	0
Ubiquitin-dependent degradation of Cyclin D1	50	3.71	17	0
Stabilization of p53	52	3.86	18	0
Ubiquitin-dependent degradation of Cyclin D	50	3.71	17	0
Antigen processing: Ubiquitination & Proteasome degradation	53	3.93	16	0
SCF-beta-TrCP mediated degradation of Emi1	54	4.01	17	0.001
Vpu mediated degradation of CD4	51	3.79	16	0.001
SCF(Skp2)-mediated degradation of p27/p21	52	3.86	17	0.001
Hedgehog ligand biogenesis	62	4.6	17	0.001
Hh mutants that do not undergo autocatalytic processing are degraded by ERAD	55	4.08	17	0.001
Hh mutants abrogate ligand secretion	55	4.08	17	0.001
p53-Dependent G1 DNA Damage Response	55	4.08	19	0.001
p53-Dependent G1/S DNA damage checkpoint	55	4.08	19	0.001

Total: number of genes in a pathway. Expected: number of expected hits according to the number of genes and miRNA belonging to the net. Hits: number of real hits.
